# High-throughput data announcement for *Escherichia coli* strain KAB04 isolated from ready-to-eat vegetable salads in Central Uganda

**DOI:** 10.1128/mra.00664-26

**Published:** 2026-08-27

**Authors:** Abubaker Kawooya, Chinyere Nkemjika Anyanwu, Emmanuel Eilu, Hussein Kafeero, Adam Ahmed Adam, Jackim Nabona, Mariam Oyedeji Amusa, Danladi Makeri, Martin Odoki, Rasheed Omotayo Adeyemo, Michael Ben Okon, Ismail Abiola Adebayo, Saheed Adekunle Akinola

**Affiliations:** 1 Department of Microbiology/Immunology, Kampala International University Western Campus 108119, Kampala, Uganda; 2 Clarke International University 127813, Kampala, Uganda; 3 Department of Mechatronics and Biomedical Engineering, Faculty of Engineering, Kyambogo University 108107, Kampala, Uganda; 4 Kampala International University 108119, Kampala, Uganda; 5 Department of Applied Sciences, School of Sciences, Nkumba University 183054, Entebbe, Uganda; 6 Department of Microbiology and Immunology, School of Medicine, Health & Life Sciences, King Ceasor University 442746, Kampala, Uganda; 7 Department of Microbiology and Parasitology, School of Medicine and Pharmacy, College of Medicine and Health Sciences, University of Rwanda 58621, Butare, Rwanda; 8 Department of Biochemistry, Faculty of Biomedical Sciences, Kampala International University 108119, Kampala, Uganda; 9 Department of Medical Biochemistry, Molecular Biology, and Genetics, School of Medicine and Pharmacy, College of Medicine and Health Sciences, University of Rwanda 58621, Butare, Rwanda; Fluxus Inc., Sunnyvale, California, USA

**Keywords:** whole-genome sequence, superbugs, resistome analysis, market-to-mouth foods, One Health, environmental contamination

## Abstract

We report the draft-genome sequence of *Escherichia coli* strain KAB04 isolated from a ready-to-eat vegetable salad in central Uganda. The assembled genome is 5,231,463 base pairs in length, with a guanine-cytosine content of 50.5%. Genome completeness is 99.19%, with a 0.63% contamination.

## ANNOUNCEMENT

*Escherichia coli* contamination of ready-to-eat (RTE) vegetable salads is a major public health concern, especially in urban low-resource settings where food safety monitoring and standard hygiene infrastructure are limited (1). These bacterial populations frequently exhibit multidrug-resistant phenotypes, contributing to severe foodborne disease outbreaks, complex clinical infections, and increased mortality (2). High-resolution genomic analysis of this bacterial pathogen is essential for detecting key genetic features linked to resistance, virulence, and mobility, which deepens insights into its biology, evolution, and public health relevance. This genome announcement presents the draft genome sequence of *E. coli* strain KAB04 isolated from a RTE vegetable salad in central Uganda (3).

Strain KAB04 was isolated from a RTE vegetable salad collected from roadside sellers in Kampala (0°18′34″N, 32°34′40″E), central Uganda (3). The RTE vegetable salad comprises shredded cabbage, carrots, cucumber, and tomatoes and is prepared without the addition of cheese or dressing. A 25 g sample of the salad was aseptically homogenized in 225 mL of sterile buffered peptone water (Oxoid, UK) using a stomacher for 2 min and incubated aerobically under static conditions at 37°C for 24 h. A loopful of the enriched culture was streaked onto MacConkey agar (Microxpress, India) and incubated overnight at 37°C under the same conditions. Presumptive pink colonies were sub-cultured on EMB agar (Condalab, Spain) and incubated under the same conditions. Colonies exhibiting the green metallic sheen characteristic of *E. coli* were selected for further characterization and genotyping (4).

Following overnight incubation of a single pure bacterial colony in 5 mL of Brain Heart Infusion (BHI) broth at 37°C, genomic DNA was extracted from the resulting culture using the QIAamp DNA Mini Kit (QIAGEN, Germany) as per the manufacturer’s instructions. Genomic DNA was quantified using a Qubit fluorometer. DNA purity was evaluated using the NanoDrop ND-1000 spectrophotometer (Thermo Fisher Scientific). Whole-genome sequencing (WGS) libraries were prepared using the Illumina DNA Prep Kit (Illumina, USA) featuring bead-linked transposomes (BLT) for simultaneous DNA fragmentation and adaptertagmentation. Tagmented gDNA was subjected to a post-tagmentation cleanup using Tagment Stop/Wash Buffer, followed by an 8-cycle PCR amplification using IDT for Illumina DNA/RNA Unique Dual (UD) Indexes to introduce sequencing cluster generation adapters. The resulting libraries were purified using a double-sided bead cleanup with Illumina Purification Beads (IPB) and eluted in resuspension buffer (RSB). Individual libraries were evaluated for fragment size distribution (average size of 600 bp) and quantified by Qubit fluorometry to determine molarity. Finally, multiplexed library pools were diluted to a 4 nM starting concentration using RSB and sequenced on the Illumina NovaSeq platform with 2 × 150 bp paired-end reads.

Raw reads were assessed for quality using FastQC (version 0.12.1) (5). Adapter sequences and low-quality bases were removed using Trimmomatic (version 0.40) (6), retaining only reads with a minimum length of 40 bp. *De novo* genome assembly was conducted using SPAdes (version 3.15.0) (7), yielding 122 contigs. Genome annotation was performed using the National Center for Biotechnology Information (NCBI) Prokaryotic Genome Annotation Pipeline (PGAP) (8). The final assembled genome has a length of 5,231,463 bp and a guanine-cytosine (GC) content of 50.5%, with an estimated completeness of 99.19% and contamination of 0.63%. A total of 5,220 genes were identified, including 4,902 predicted protein-coding genes (Table 1). All software tools were run using default parameters unless otherwise specified.

**TABLE 1:** Features of annotated *Escherichia coli* KAB04

Parameters	Value
Genome size	5,231,463 bp
Total number of genes	5,220
Protein-coding genes	4,902
Total number of CDSs	5,138
Hypothetical CDS	613
Number of scaffolds	115
Number of contigs	122
Scaffold *N*_50_	140.1 kb
Scaffold *L*_50_	12
Contig *N*_50_	115.9 kb
Contig *L*_50_	13
GC%	50.5%
Genome coverage	50.0x
Completeness (%)	99.19
Contamination (%)	0.63

## Data Availability

This draft Genome Shotgun project has been deposited in NCBI GenBank under accession number JBYKMT000000000 (9) associated with BioProject PRJNA1467415 (10) and BioSample SAMN60244552 (11).
